# Short Link N Modulates Inflammasome Activity in Intervertebral Discs Through Interaction with CD14

**DOI:** 10.3390/biom14101312

**Published:** 2024-10-16

**Authors:** Muskan Alad, Michael P. Grant, Laura M. Epure, Sunny Y. Shih, Geraldine Merle, Hee-Jeong Im, John Antoniou, Fackson Mwale

**Affiliations:** 1 Department of Surgical and Interventional Sciences, McGill University, Montreal, QC H3T 1E2, Canada; 2 Orthopaedic Research Laboratory, Lady Davis Institute for Medical Research, Montreal, QC H3T 1E2, Canada; 3 SMBD-Jewish General Hospital, McGill University, Montreal, QC H3T 1E2, Canada; 4 Faculty of Medicine, McGill University, Montreal, QC H3T 1E2, Canada; 5 Chemical Engineering Department, Polytechnique Montréal, Montreal, QC H3C 3A7, Canada; 6 Department of Bioengineering, University of Illinois Chicago, Chicago, IL 60612, USA

**Keywords:** intervertebral disc, degeneration, back pain, link N, inflammasome, interleukin-1ß

## Abstract

Intervertebral disc degeneration and pain are associated with the nucleotide-binding domain, leucine-rich repeat, and pyrin domain-containing 3 (NLRP3) inflammasome activation and the processing of interleukin-1 beta (IL-1β). Activation of thehm inflammasome is triggered by Toll-like receptor stimulation and requires the cofactor receptor cluster of differentiation 14 (CD14). Short Link N (sLN), a peptide derived from link protein, has been shown to modulate inflammation and pain in discs in vitro and in vivo; however, the underlying mechanisms remain elusive. This study aims to assess whether sLN modulates IL-1β and inflammasome activity through interaction with CD14. Disc cells treated with lipopolysaccharides (LPS) with or without sLN were used to assess changes in Caspase-1, IL-1β, and phosphorylated nuclear factor kappa-light-chain-enhancer of activated B cells (NFκB). Peptide docking of sLN to CD14 and immunoprecipitation were performed to determine their interaction. The results indicated that sLN inhibited LPS-induced NFκB and Caspase-1 activation, reducing IL-1β maturation and secretion in disc cells. A significant decrease in inflammasome markers was observed with sLN treatment. Immunoprecipitation studies revealed a direct interaction between sLN and the LPS-binding pocket of CD14. Our results suggest that sLN could be a potential therapeutic agent for discogenic pain by mitigating IL-1β and inflammasome activity within discs.

## 1. Introduction

Chronic low back pain (LBP) is a prevalent and under-served condition, ranking in the top five reasons for doctor visits, surgeries, and hospitalization worldwide [1,2,3,4,5,6]. The World Health Organization estimates that about one-third of the global population suffers from back pain, with a lifetime prevalence of over 60% [1,2]. A primary cause of LBP is intervertebral disc degeneration (IVDD), characterized by extracellular matrix degradation, inflammation, endplate sclerosis, cell death, and hyperinnervation of nociceptive nerve fibers, leading to persistent pain [4,5,6].

The intervertebral disc (IVD) is an avascular fibro-cartilaginous tissue comprising a peripheral collagen-rich lamellae annulus fibrosus (AF), a central gelatinous proteoglycan nucleus pulposus (NP), and superior and inferior cartilaginous endplates (CEPs). The avascular nature of the IVD limits its ability to repair and regenerate [4].

In a normal IVD, sensory nerve fibers are typically confined to the outermost layers of the AF [7,8,9]. During tissue injury or inflammation, these neurons are thought to transmit nociceptive information related to pain [7,10,11,12]. In degenerative human IVDs, there is an observed increase in sensory innervation within the inner AF layers in response to signalling molecules called neurotrophins (NTs) [13]. These NTs, produced in the IVD, are transported retrogradely to the neurons, where they maintain neuronal survival and stimulate the expression of nerve growth factors (NGFs) and neurotransmitters associated with nociception [14,15,16]. NGFs are the major player in maintaining the survival of sensory neurons that innervate IVDs. In an inflammatory environment, NGF expression increases and is upregulated by cytokines such as Interleukin (IL)-1β, which are highly expressed in painful discs [17,18,19].

One of the main factors causing the activation of IL-1β is the canonical NLR pyrin domain-containing 3 (NLRP3) inflammasome [20]. This intracellular protein complex plays an important role in innate immunity by activating Caspase-1 and processing IL-1β, along with other pro-inflammatory cytokines, thus contributing to discogenic pain. Activation of the inflammasome is triggered by pattern recognition receptors (PRRs), specifically Toll-like receptors (TLRs), which directly recognize molecular structures such as pathogen-associated molecular patterns (PAMPs) or damage-associated molecular patterns (DAMPs). Among the TLRs expressed in disc cells, TLR4 is notably associated with discogenic pain, mediating inflammation through ligand recognition. To enhance ligand-binding efficiency, TLR4 requires CD14, an extracellular cofactor receptor [21]. This glycoprotein is present in both membrane-anchored and soluble forms [21] and interacts with a multitude of ligands, including lipopolysaccharide (LPS) and other PAMPs and DAMPs, increasing ligand-binding affinity to TLR4 [21,22].

Despite the understanding of these mechanisms, a gold-standard treatment for discogenic pain remains elusive, and targeted therapies to decrease hyperinnervation in degenerative disc disease are lacking [23]. Short Link N (sLN), a peptide derived from the N-terminal region of the link protein, has shown potential as a therapeutic agent. Our group was the first to demonstrate the anabolic effects of sLN on the IVD [24,25,26,27,28]. We and others have shown that it can stimulate the synthesis of proteoglycans and collagen in articular cartilage and IVD tissues [26,27,28,29,30,31,32]. Importantly, as IVDD and acute injury are associated with local inflammation, the ability of sLN to stimulate the expression of extracellular matrix proteins and downregulate the expression of degrading metalloproteinases is maintained in an inflammatory milieu. In a rabbit disc puncture model, we have shown that sLN can regenerate disc tissue and restore disc height [26]. In addition to the anabolic effects of sLN on matrix homeostasis, we also discovered the potential role of sLN in modulating pain behaviour in vitro [16] and in vivo, 6 h post-administration in a Partial Medial Meniscectomy (PMM) murine model with advanced OA [26].

This study builds upon previous research by examining the effects of sLN on inflammasome activity in the IVD, focusing on its interaction with CD14. We hypothesize that sLN inhibits NLRP3 activation in NP cells through CD14 interaction, preventing disc degeneration progression. By elucidating these mechanisms, our research aims to advance therapeutic strategies for discogenic pain and potentially other inflammatory conditions.

## 2. Materials and Methods

### 2.1. Peptide Synthesis

sLink N (DHLSDNYT) was synthesized with a purity >98% by CanPeptide (Pointe Claire, QC, Canada).

### 2.2. Antibodies

Anti-interleukin-1ß antibody was purchased from ThermoFisher Scientific (Waltham, MA, USA; Cat# MM425B), anti-6x-His Tag from ThermoFisher Scientific (cat# MA1-21315), anti-NLRP3 from MilliPore Sigma (Burlington, MA, USA (Cat# SAB5700723), anti-GAPDH from Millipore Sigma (cat# G8795), anti-CD14 from Abcam (Cambridge, UK; cat# ab183322), anti-Caspase-1 from Abcam (cat# ab286125), and anti-phospho-NF-κB p65 (Ser536) antibody from Cell Signaling Technology (Danvers, MA, USA; cat# 3033). Anti-sLN antibody was custom-made by ThermoFisher.

### 2.3. Human Nucleus Pulposus Cell

Human NP (hNP) cells were purchased from ScienCell Research Laboratories (Carlsbad, CA, USA, cat. #4800). Cells were cultured and maintained in PrimeGrowth^®^ Culture Medium (Wisent Bioproducts, cat# 319-510). Different lots were purchased and considered to be different donors. Medium was replaced every three days. Cells were used up to passage four.

### 2.4. Murine Macrophages

Murine RAW 264.7 macrophage cells were purchased from Sigma-Aldrich CO, (St. Louis, MO, USA; cat. #91062702). Cells were cultured and maintained in DMEM supplemented with 10% FBS/FCS (Wisent Bioproducts, cat# 319-510). Medium was replaced every three days. Cells were used until passage four.

### 2.5. Rabbit Annular Puncture Model of IVDD

Six New Zealand White rabbits (specific pathogen-free (SPF), 4–6-month-old skeletally mature rabbits, Western Oregon Rabbit Co., Philomath, OR, USA) weighing approximately 3.5 kg were used in the present study with the approval of the Institutional Animal Care and Use Committee, La Jolla, CA, USA. IVD degeneration was induced in all rabbits as previously described [26]. Briefly, the rabbits were placed in a lateral prone position and the anterior surfaces of three consecutive lumbar IVDs (L2/3, L3/4 and L4/5) were exposed through a posterolateral retroperitoneal approach by blunt dissection of the psoas muscle. IVD degeneration was induced at the L2/3 and L4/5 levels by AF puncture in the ventral aspect into the NP using an 18G needle with a stopper device that allows the needle to penetrate to a maximum depth of 5 mm. The IVD at the level L3/4 was left intact and used as a non-punctured control. The surgical wound was repaired in layers and the skin was closed using staples and IVD degeneration was confirmed by X-rays at 2 weeks post-surgery.

Two weeks post-surgery, both punctured discs (L2/3 and L4/5) of each rabbit were injected intradiscally through their anterolateral surfaces into the NP area with either saline (10 μL/disc) or 25 μg sLN dissolved in 10 μL saline, using a MS*GFN25 micro-syringe equipped with a XX*MS16 needle (Ito Corporation, Fuji, Japan) [26]. The non-punctured disc (level L3/4) of each rabbit remained untreated and was used as an internal control. Rabbits were closely monitored for behaviour, appetite, and change in body weight for the duration of this study. At 12 weeks after treatment, rabbits in each group were anesthetized with ketamine hydrochloride (25 mg/kg) and acepromazine maleate (1 mg/kg) and euthanatized with an excess dose of pentobarbital (Euthanasia B solution: Henry Schein Inc., Melville, NY, USA). Rabbit discs were excised from spines and prepared for histological evaluation.

### 2.6. Histology

The excised rabbit IVD specimens, inclusive of adjacent vertebral segments, underwent fixation in 10% neutralized formalin and decalcification with Cal-Ex™ II Fixative/Decalcifier and were subsequently paraffin-embedded. Sagittal sections of 4 μm thickness were prepared from each IVD, dewaxed using Safeclear™ Tissue Clearing Agent, and stepwise rehydrated in ethanol 100%, 95%, 70%, 50%, and distilled water. Sections were further processed using Vectastain Elite ABC-HRP Peroxidase kit (Vector Laboratories, Newark, CA, USA; cat# PK-6100) and probed with anti-NLRP3 antibody [1:100], specific antibodies targeting Protein Gene Product 9.5 (PGP9.5) and nerve growth factor (NGF) for neuronal innervation, and NLRP3 to mark inflammasome activation. Following staining, sections were dehydrated and mounted using Permount™ (ThermoFisher Scientific, cat# SP15-100) for preservation. Images were captured using a Leica DM LB2 light microscope. Image analysis to determine NLRP3 expression was performed using ImageJ Software (ver 1.53K).

### 2.7. Inflammasome Signaling

Micropellets of hNP cells were prepared by centrifuging 500,000 hNP cells at 400 rcf for 10 min in a microcentrifuge tube. Cells were subjected to overnight starvation in a serum-free PrimeGrowth Disc Cell medium. For the dose–response signalling, LPS [1 μg/mL] was preincubated for 10 min with the indicated concentrations of sLN [0.05, 0.5, 5.0 μg/mL] and administered to the cells for 45 min. For the temporal effect, LPS [1 μg/mL] was preincubated for 10 min with sLN [0.5 μg/mL] and administered to the cells for 10, 30, 60, 120, and 180 min. The incubations were by lysing the cells in RIPA buffer (Millipore Sigma, cat# R0278) containing protease (Millipore Sigma, cat#’s 535142, 535140) and phosphatase (Millipore Sigma, cat# 524627) inhibitors. Lysate was electrophoresed by Western blotting on a 4–20% Tris-Glycine polyacrylamide gel (ThermoFisher Sientific, cat# XP04200) and transferred to PVDF membrane (BioRad, Hercules, CA, USA; cat# 1620177XTU). Blots were blocked in BSA and incubated with anti-P-NF-κB antibody to monitor for inflammasome activation. Blots were subsequently incubated with anti-mouse or anti-rabbit conjugated horseradish peroxidase (HRP) secondary antibodies (Jackson ImmunoResearch, Westgrove, PA, USA; cat#’s 115-035-166 and 111-035-144, respectively). Blots were washed and incubated with Amersham ECl Select Western blotting detection reagent (Millipore Sigma, cat# GERPN2235) and imaged using a BioRad VersaDoc Imaging system (BioRad). The dose and temporal effects of LPS, CD14, and sLN on inflammasome activation were monitored by measuring P-NF-κB from 0 to 180 min.

### 2.8. Assessment of Inflammasome Markers in hNP Cells

hNP cells were cultured as micropellets containing 500,000 cells. Micropellets were incubated for 48 h in 0.5 mL of medium supplemented with either 1.0 μg/mL LPS, a combination of 0.5 μg/mL sLN + LPS or just medium as a control group. The reaction was terminated by washing pellets in ice-cold PBS and immediately processing for RNA extraction.

### 2.9. Caspase-1 and IL-1β Maturation

hNP cells were cultured as micropellets and incubated with LPS [1.0 μg/mL] with or without sLN [0, 0.05, 0.5 or 5.0 μg/mL] for 48 hrs. The reaction was terminated by washing pellets in cold PBS and lysing cells in RIPA buffer containing protease inhibitors. Lysate was electrophoresed by Western blotting on a 4–20% Tris-Glycine polyacrylamide gel and transferred to PVDF membranes. Blots were blocked in BSA and incubated with anti-Caspase-1 [1:1000] or anti-IL-1β [1:1000] antibodies. Blots were washed and incubated with secondary antibodies followed by detection reagent and imaging on a BioRad VersaDoc imaging system.

### 2.10. RNA Extraction and Quantitative Real-Time PCR

RNA extraction was performed using a Total RNA mini-kit (Geneaid Biotech Ltd., New Taipei City, Taiwan) following the guidelines provided by the manufacturer. This RNA served as a template for the synthesis of complementary DNA (cDNA) using superscript Vilo cDNA synthesis kit (Thermo Fisher Scientific, Waltham, MA, USA). Quantitative real-time PCR (qRT-PCR) was employed to assess the gene expression levels of key inflammasome components, *NLRP3*, *PYC*, *CASP1*, *IL1B*, *TNFA* (Table 1), macrophage polarization markers, *CD80*, *CD86*, *IL1B*, *ARG*, *CD206*, *IL10* (Table 2), and *GAPDH*. CYBR green master mix (Thermo Fisher Scientific, Waltham, MA, USA) was used for detection using an ABI 7500 fast light cycler (Applied Biosystems, Waltham, MA, USA). Gene expression was normalized to *GAPDH* as the reference gene. Comparative Ct was used to measure gene expression using the equation

Relative expression = 2^−ΔΔCt^

Relative expression values were normalized to control values to compare for changes in gene expression.

### 2.11. Macrophage Polarization Induced by hNP

RAW 264.7 were seeded in 6-well plates (250,000 cells/well) in DMEM supplemented with 10% FBS and 1% Pen-Strep. Cell inserts (0.4 μm) were added into the wells (Sarstedt, Germany; cat# 83.3939.040) and hNP cells were seeded in the inserts (500,000 cells/insert) in PrimeGrowth medium supplemented with 10% FBS and 1% Pen-Strep. The hNP cells in the inserts were then incubated with LPS [1.0 μg/mL] with or without sLN [0.5 μg/mL] for 48 h. The incubation was terminated by washing RAW cells with ice-cold PBS and processing for RNA extraction.

### 2.12. Immunoprecipitation

Protein A/G agarose beads (ThermoFisher Scientific; cat# 20421) were incubated with anti-His antibody. Following washing, anti-His bound Protein A/G agarose beads were incubated with His-tagged CD14 [0.25 μg] (Abcam, cat# ab167706) with or without sLN [1.0 μg] for 1 h. Beads were washed and processed for dot-blot probing by boiling for 5 min in Laemmli buffer with DTT. Blots were washed and probed with anti-sLN antibody [1:500] followed by goat anti-rabbit HRP-conjugated secondary antibody and detection with ECL Select. Dot-blots were imaged on a BioRad VersaDoc imager. Competitive IP was performed by loading Avidin-labelled agarose beads (ThermoFisher Scientific; cat# 20219) with 1.0 μg biotinylated LPS (LPS-Biotin) (InvivoGen, San Diego, CA, USA; cat# tlrl-lpsbiot). Beads were then washed in PBS and incubated with CD14 [0.25 μg] with or without unlabeled LPS [1.0 μg] or sLN [0.5 μg] for 1 hr. Beads were subsequently washed in PBS and processed for Western blotting. Samples were electrophoresed by Western blotting on a 4–20% Tris-Glycine polyacrylamide gel and transferred to PVDF membrane. Blots were blocked in BSA and incubated with anti-CD14 antibody. Blots were subsequently incubated with anti-mouse conjugated-HRP secondary antibody. Blots were washed and incubated with Amersham ECl Select Western blotting detection reagent and imaged using a BioRad VersaDoc Imaging system (Bio-Rad, Hercules, CA, USA).

### 2.13. Statistical Analysis

Data were analyzed by ANOVA followed by a post hoc Dunnett’s test. A *p*-value of less than 0.05 was considered statistically significant.

## 3. Results

### 3.1. Impact of sLN on NLRP3 Expression in a Rabbit Annular Puncture Model of IVDD

**Figure 1:**
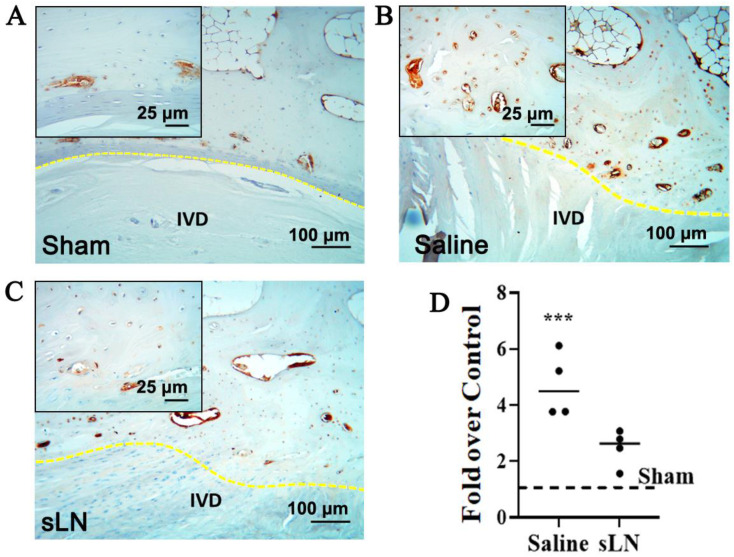
Immunohistochemistry of NLRP3 expression in a degenerative IVD model. Rabbit IVDs from a puncture model were given a single injection of either saline or sLN. Sham animals were used as controls. Twelve weeks following injection, IVDs were processed for immunohistochemistry and the detection of NLRP3. Higher-magnification inset images are included and demarcated to highlight specific areas of interest. Immunohistochemistry of NLRP3 in (**A**) sham and degenerative IVDS treated with (**B**) saline or (**C**) sLN. (**D**) Densitometry of NLRP3 expression in IVDs presented in (**A**). Statistical significance was assessed using Student’s *t*-test (comparison between saline and sLN); ***, *p* < 0.001; *n* = 4. (IVD—intervertebral disc, NLRP3—nucleotide-binding domain, leucine-rich repeat, and pyrin domain-containing 3, yellow dotted line—endplate).

Given the role of NLRP3 inflammasome activation in IVDD [33,34,35], immunohistochemistry analysis was performed to assess the presence and potential activation of NLRP3 in a rabbit annular puncture model of IVDD (Figure 1). In this model, sLN has previously been shown to increase disc height significantly by restoring proteoglycan and collagen content.

Figure 1A–C include also an inset image that highlights the specific areas of NLRP3 expression within the IVD tissue. The inset provides a higher magnification view of the regions of interest, allowing for detailed observation of the differential expression patterns of NLRP3 between saline-treated and sLN-treated discs. This visual representation underscores the modulatory effect of sLN on inflammasome activation. In the control IVDs, NLRP3 was minimally detected (Figure 1A–D) highlighting a baseline level of expression. In contrast, in IVDs subjected to puncture and treated with saline, a substantial upregulation of NLRP3 expression was observed, especially in areas surrounding the cartilaginous and bony endplates (Figure 1B–D). This upregulation indicates an inflammatory response possibly linked to physical disruption and saline treatment. Interestingly, the application of sLN to punctured discs markedly attenuated NLRP3 expression, aligning it closely with the levels seen in sham IVDs (Figure 1A,C,D). This suggests that sLN treatment not only mitigates the inflammatory response marked by NLRP3 activation but also mirrors a state similar to untreated, healthy IVDs. The differential expression patterns of NLRP3, observed through comparative analysis of sham, vehicle-treated, and sLN-treated discs, underscore the potential of sLN as a modulator of inflammasome-mediated pathways in the context of disc degeneration.

### 3.2. Regulation of Inflammasome Signaling by sLN in Human Nucleus Pulposus Cells

**Figure 2:**
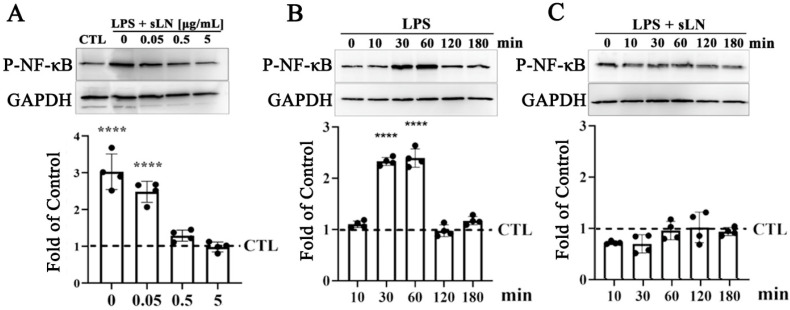
Evaluation of NF-κB activation in human nucleus pulposus cells treated with sLN and LPS. (**A**) Human nucleus pulposus (NP) cells were treated with LPS alone or in combination with sLN with the indicated concentrations (0.05, 0.5, or 5.0 μg/mL) for 45 min. Western blotting was performed to detect activation of P-NF-κB. Blots were normalized to GAPDH for loading by densitometry and calculated as a fold increase over control. The densitometry of the blots is presented in the graph below. (**B**) HNP cells were incubated with LPS alone or (**C**) LPS and 0.5 μg/mL sLN for the indicated times (0–180 min). P-NF-κB signal was normalized to GAPDH and calculated as a fold-over control. Statistical significance was assessed using ANOVA and post hoc Dunnett’s test (comparison to control); ****, *p* < 0.0001; *n* = 4. Original Western blot images are available in Supplementary Materials.

To explore the potential of sLN in modulating LPS-induced inflammasome activation, we assessed the activation of NFκB, a central component of the canonical TLR signalling pathway [3,36,37] in hNP cells (Figure 2).

Exposure to LPS for 30 min led to a significant increase in NFκB phosphorylation (P-NFκB), with Western blot analysis revealing a threefold enhancement in *p*-NFκB levels (Figure 2A). Notably, the concurrent administration of sLN with LPS exhibited a dose-responsive attenuation in P-NFκB levels. Specifically, at concentrations of 0.5 and 5 μg/mL, sLN effectively reduced the P-NFκB signal to levels that were not significantly different from the untreated control, suggesting a potent inhibitory effect on NFκB activation. Further investigation into the time-dependent effects of sLN on LPS-stimulated NFκB activation revealed that NFκB phosphorylation peaked at 30 and 60 min post-LPS treatment in hNP cells (Figure 2B). Interestingly, co-treatment with 0.5 μg/mL sLN and LPS significantly mitigated the P-NFκB signal, indicating the effective suppression of LPS-induced NFκB activation by sLN. This reduction points to the capacity of sLN to modulate inflammatory signalling pathways, highlighting its therapeutic potential in conditions characterized by inflammasome overactivation.

### 3.3. Modulation of Inflammasome Activation Markers by sLN

To explore the regulatory effect of sLN on inflammasome activation markers [20,38,39,40,41], hNP cells were subjected to a 48 h treatment with LPS alone and in combination with sLN.

**Figure 3:**
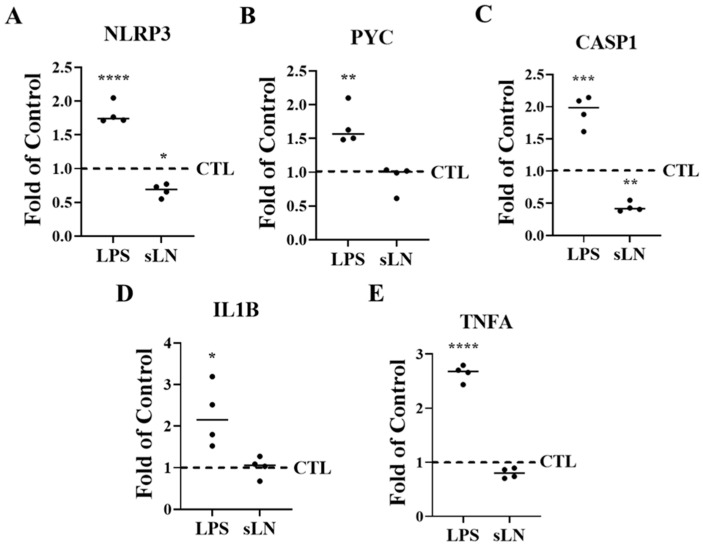
Suppression of inflammasome markers by sLN. HNP cells were incubated for 48 h in medium supplemented with LPS or LPS and 0.5 μg/mL sLN. Control samples were incubated with medium alone. RNA expression is shown for (**A**) *NLRP3*, (**B**) *PYC*, (**C**) *CASP1*, (**D**) *IL1B*, and (**E**) *TNFA*. Treatments were compared to controls. ANOVA and post hoc Dunnett’s test; *, *p* < 0.05; **, *p* < 0.01; ***, *p* < 0.001; ****, *p* < 0.0001; *n* = 4.

The analysis revealed that LPS stimulation significantly increased the expression levels of genes critical to inflammasome assembly, specifically *NLRP3* and *PYCARD*, compared to untreated controls. However, the introduction of sLN alongside LPS noticeably tempered this upregulation, indicating an inhibitory effect of sLN on these inflammasome components (Figure 3A,B). Additionally, the LPS-driven increases in key inflammatory markers—*Caspase-1*, *IL-1β*, and *TNF-α*—were effectively mitigated upon co-treatment with sLN, further underscoring the capacity of sLN to blunt inflammasome-related responses (Figure 3C–E).

### 3.4. Combined Inhibition of Caspase-1 Activation and IL-1β Secretion by sLN in LPS-Stimulated

**Figure 4:**
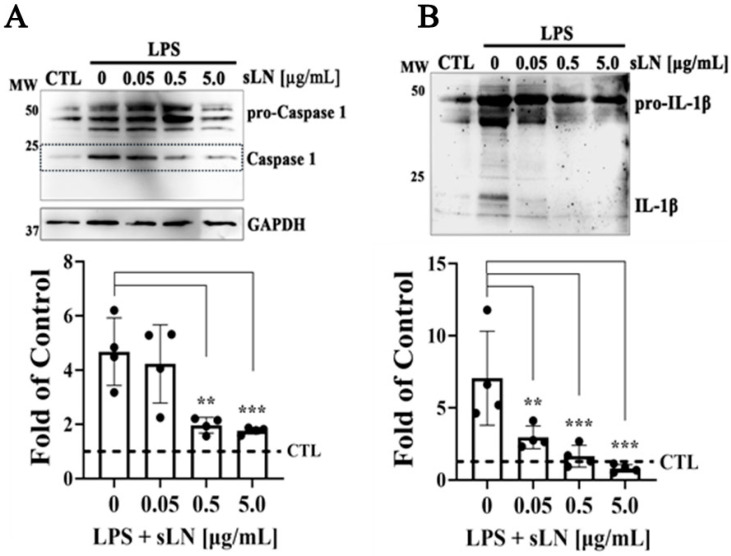
Assessment of Caspase-1 activation and IL-1β secretion in hNP cells. HNP cells were treated with LPS alone or in combination with sLN at varying concentrations (0.05, 0.5 and 5.0 μg/mL) for 48 h. (**A**) Western blotting and densitometry of Caspase-1 demonstrating Pro-Caspase-1, Caspase-1, and GAPDH as loading control. (**B**) Western blotting and densitometry of pro- and mature forms of IL-1β. Data are expressed as fold-over controls. Statistical significance was assessed using ANOVA and post hoc Dunnett’s test (treatments compared to LPS alone); **, *p* < 0.01; ***, *p* < 0.001; *n* = 4. Original Western blot images are available in Supplementary Materials.

In this section, we discuss the dual modulatory effects of sLN on key inflammatory pathways in hNP cells (Figure 4). Given the central role of Caspase-1 in the inflammatory cascade, particularly in processing and maturing IL-1β, its regulation is crucial for mitigating inflammation-related pathologies in IVDD [33,42].

hNP cells were stimulated with LPS to induce an inflammatory state, subsequently moderated by treatment with sLN in a dose-dependent manner. Consistent with established inflammatory pathways, LPS stimulation resulted in a significant increase in both Caspase-1 activation and IL-1β secretion (Figure 4A,B), marking an active inflammasome response. This observation underscores the role of LPS in triggering cellular inflammation, relevant to IVDD mechanisms. Remarkably, sLN treatment demonstrated a dose-dependent mitigation of both inflammatory markers. Specifically, at 0.5 μg/mL and 5.0 μg/mL, sLN significantly decreased Caspase-1 activation and IL-1β secretion. This suppression suggests the capacity of sLN to directly modulate inflammasome signalling, reducing the subsequent inflammatory output. The inhibitory impact on Caspase-1 activation and IL-1β secretion was most pronounced at the highest sLN concentration (5.0 μg/mL), indicating a potent anti-inflammatory effect that scales with dosage. This dose-dependent relationship further bolsters the potential of sLN as a therapeutic agent, offering insight into optimal dosing strategies for inflammation attenuation within the IVD environment. The findings presented in Figure 4 outline the dual modulatory impact of sLN on key inflammatory pathways within hNP cells incubated with LPS. By significantly downregulating Caspase-1 activation and IL-1β secretion, sLN asserts itself as a promising candidate for developing treatments aimed at counteracting the inflammatory processes at play in IVDD.

### 3.5. Inflammasome Activation and Macrophage Polarization

**Figure 5:**
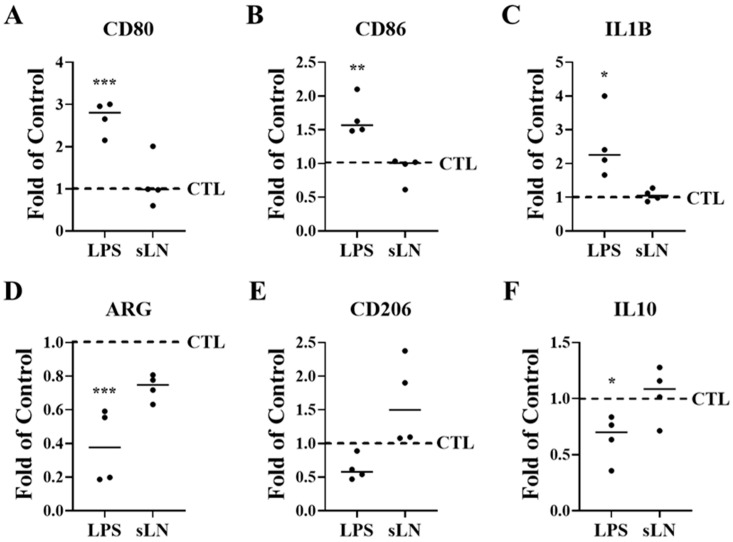
Inflammasome activation and hNP-induced macrophage polarization. RAW macrophages and hNP cells were co-cultured to determine the effects of hNP on macrophage polarization. HNP cells were stimulated with LPS and incubated for 48 hrs with RAW cells. Expression of M1 (**A**–**C**) and M2 (**D**–**F**) markers in RAW macrophages were measured by qPCR. Plots represent fold-over control. ANOVA and post hoc Dunnett’s test (treatments compared to control); *, *p* < 0.05; **, *p* < 0.01; ***, *p* < 0.001; *n* = 4.

Degenerative IVD cells release pro-inflammatory cytokines such as IL-1β, which initiate the recruitment of immune cells, thereby triggering an inflammatory cascade [3,34,35,43]. To assess whether inflammasome activation in hNP cells influences immune cell activation, we conducted co-culture experiments with RAW macrophages. Exposure of hNP cells to LPS resulted in a significant upregulation in the expression of *CD80*, *CD86*, and *IL1B*, markers indicative of M1 polarization, which is associated with an inflammatory macrophage phenotype; these findings are illustrated in Figure 5A–C.

In contrast, the expression levels of arginase (*ARG*) and *IL10*, which are markers of the M2 macrophage phenotype involved in tissue repair, were significantly reduced (Figure 5D–F) following LPS incubation. Additionally, the expression of the M2 marker *CD206* showed a decreasing trend.

When hNP cells were co-incubated with sLN and LPS, the levels of M1 polarization markers did not differ significantly from those in the control group (Figure 5A–C), indicating that sLN mitigates the LPS-induced M1 polarization of RAW macrophages. Furthermore, the expression levels of M2 polarization markers in these macrophages remained stable following co-treatment with sLN and LPS, showing no significant changes compared to control conditions (Figure 5D–F). These data suggest that sLN can modulate macrophage polarization, potentially attenuating the pro-inflammatory response while maintaining tissue repair processes.

### 3.6. Interaction of sLN with CD14

**Figure 6:**
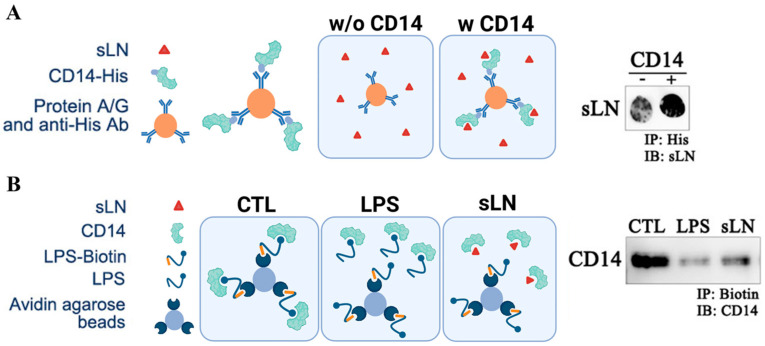
Interaction and immunoprecipitation of sLN and CD14. (**A**) Schematic on the co-immunoprecipitation of CD14 and sLN and dot-blot showing enrichment of sLN following pull-down with CD14. (**B**) Schematic demonstrating the competitive co-immunoprecipitation of biotinylated LPS for CD14 in the presence of unlabeled LPS and sLN. Western blot showing the detection of CD14 following the indicated pull-downs. Lanes from left to right: control (CD14 only), LPS-treated, sLN-treated. Original Western blot images are available in Supplementary Materials.

To determine whether sLN can interact directly with CD14, we conducted a series of immunoprecipitation experiments. As shown in Figure 6A, we observed an enrichment of sLN peptide on our dot-blots when we performed pull-down experiments with CD14 and sLN. In the absence of CD14, minimal sLN reactivity to an anti-sLN antibody was observed (Figure 6A). In addition to a direct interaction with CD14, we performed competitive co-IP experiments to determine whether sLN can compete with the binding and pull-down of CD14. As shown in Figure 6B, in control experiments using biotinylated-LPS (LPS–biotin), we were able to effectively co-IP CD14 (lane CTL). However, when LPS–biotin was incubated with either unlabeled LPS (lane LPS) or sLN (lane sLN), the pull-down of CD14 was considerably reduced (Figure 6B).

### 3.7. Schematic Mechanisms of sLN Inhibition of Inflammasome Activation in IVD Cells

**Figure 7:**
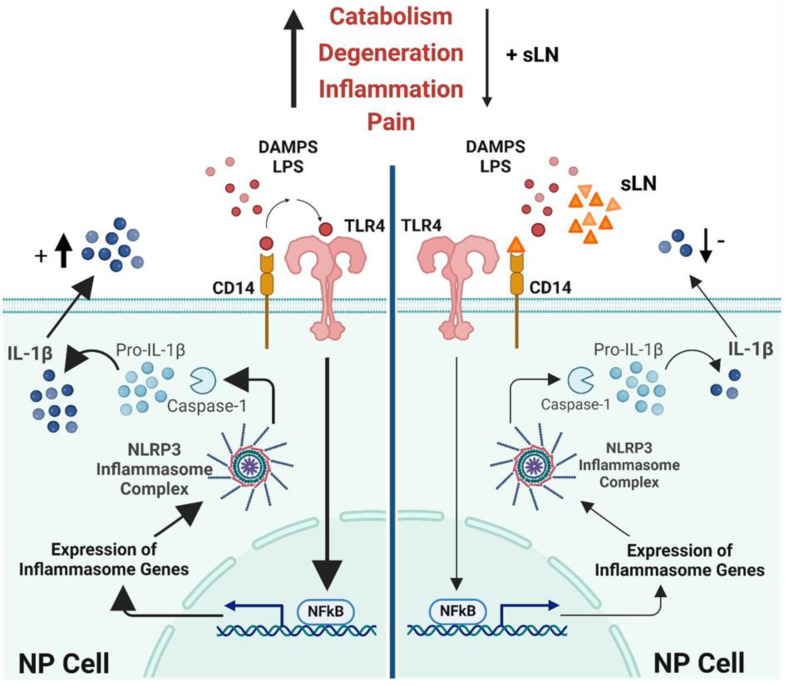
Mechanism of sLN inhibition of inflammasome activation in disc cells. This schematic illustrates the process by which sLN inhibits inflammasome activation in nucleus pulposus cells, detailing the interactions and molecular pathways involved.

Figure 7 provides a comprehensive illustration of the biochemical pathways and molecular interactions through which the peptide sLN modulates inflammasome activity in NP cells, a key component of intervertebral disc tissue. This visual representation is designed to elucidate the anti-inflammatory mechanisms of sLN with a focus on its potential therapeutic implications for treating IVDD. NP cells are shown to contain the inflammasome complex, primarily consisting of NLRP3 and pro-Caspase-1. When activated, the inflammasome plays a pivotal role in the maturation and secretion of IL-1β, a pro-inflammatory cytokine critically involved in the pathogenesis of IVDD.

#### 3.7.1. Pathway of Activation and Inhibition

The schematic details the activation pathway starting from the cell surface, where TLR4 interacts with LPS, leading to the activation of NFκB via the MyD88-dependent pathway. NFκB translocates to the nucleus, promoting the transcription of pro-inflammatory genes, including those encoding pro-IL-1β. This precursor is processed by Caspase-1, activated within the inflammasome complex, culminating in the release of active IL-1β.

#### 3.7.2. Role of sLN

The key feature of the schematic is the depiction of the inhibitory action of sLN. sLN is shown binding to CD14, a co-receptor involved in TLR4 signalling, which effectively interrupts the cascade that leads to NFκB activation and subsequent IL-1β production. This interference is illustrated by a reduction in the expression of inflammasome genes and decreases in the maturation of Caspase-1 and IL-1β through regulation of the canonical inflammasome signal transduction pathway. Additionally, potential direct interactions of sLN with components of the inflammasome itself are possible, suggesting multiple points of therapeutic intervention.

#### 3.7.3. Outcome

The ultimate effect of sLN’s interaction is portrayed as a significant reduction in IL-1β secretion, leading to decreased inflammatory responses within the disc environment. This is visually represented by fewer IL-1β molecules being released from the cells, indicating effective suppression of inflammation.

This detailed visual guide not only illustrates the complex interactions involved in sLN-mediated inhibition of inflammasome activation but also highlights the therapeutic potential of sLN in mitigating inflammation-driven disc degeneration, offering insights into future clinical applications.

## 4. Discussion

This study aimed to assess whether sLN inhibits NLRP3 activation and IL-1β secretion in NP cells and modulates inflammasome activity through interaction with CD14, subsequently inhibiting IVD inflammation and hyperinnervation. Moreover, recent studies have demonstrated that the inflammatory cascade triggered by the increased synthesis of the inflammatory cytokines in the IVD stimulates macrophages via crosstalk into classically activated macrophages (M1) [3]. This mechanism intensifies the response by promoting inflammation and extracellular matrix degradation within the IVD, initiating a harmful cycle of matrix breakdown that further drives the progression of IVDD and alternatively activates macrophages (M2) involved in tissue remodelling and immunomodulatory functions [3,44]. Therefore, this study also explores the effects of sLN on macrophage polarization.

Our results demonstrate that sLN significantly inhibits the activation of key inflammasome markers such as NFκB, Caspase-1, and IL-1β in hNP cells when these cells are treated with LPS. This inhibition is evident through Western blot and RT-PCR analysis, which showed reduced levels of pro-inflammatory cytokines and inflammasome components upon treatment with sLN. Through peptide docking studies using the crystal structure of CD14 (4glp) and the CABS-dock web server (http://biocomp.chem.uw.edu.pl/CABSdock/; 12 July 2022), we identified the potential of sLN to interact with CD14. Interestingly, using the same docking server, we did not obtain any hits for an interaction with sLN and TLR4. The interaction between sLN and CD14 was substantiated by immunoprecipitation experiments, suggesting that sLN may modulate inflammasome activity through direct interaction with CD14, which is critical in the TLR4 signalling pathway. Further studies, possibly involving molecular dynamics simulations or mutagenesis, could provide deeper insights into the mechanistic details of this interaction. It was also found that treatment with sLN could prevent the typical M1 polarization of macrophages induced by LPS, maintaining a more balanced M1/M2 macrophage phenotype [45,46,47,48]. This indicates a broader anti-inflammatory role of sLN in immune modulation [49,50].

This study builds upon the established understanding that the NLRP3 inflammasome plays a significant role in the pathophysiology of IVDD [51,52]. Several studies have highlighted a detailed contribution of inflammasome activation to cytokine maturation and secretion. Our findings on the inhibitory effects of sLN on key components like NFκB, Caspase-1, and IL-1β align with these insights [40,51,52]. However, unlike previous research, which largely focused on the consequences of inflammasome activity [40,41,42,52,53,54], the present study introduces a novel peptide, sLN, that interacts directly with CD14 to modulate its activity, suggesting the inhibition of the regulatory pathway of NLRP3. This interaction is particularly significant because it implicates a more targeted mechanism of inflammasome regulation, which has not been extensively explored in earlier studies. Additionally, prior studies have often highlighted the challenge of effectively targeting the inflammation cascade in IVDD without affecting other physiological processes [55,56]. The capacity of sLN in binding to CD14 and modulating the response to LPS, as demonstrated in our docking and immunoprecipitation experiments, addresses this gap by offering a potential method to dampen harmful inflammation while preserving necessary immune functions selectively.

Our findings also add a layer of complexity to the understanding of macrophage polarization in the context of IVDD. Several studies have demonstrated that disc degeneration impacts the structure and function of spinal muscles. Changes in the spinal muscles, such as multifidus, may detrimentally affect spinal flexibility and exacerbate pain [57,58,59]. Moreover, macrophages appear to play a role in degenerative processes within spinal muscles [58], underscoring their multifaceted role in both IVDD and associated muscle changes. This highlights the potential for therapeutic strategies targeting macrophage polarization to address these interconnected pathologies. Our study is one of the few that demonstrate the ability of a specific peptide to maintain a balanced macrophage response under inflammatory conditions, which could lead to new approaches to managing IVDD.

The implications of these findings are twofold. Firstly, sLN represents a promising therapeutic candidate for the treatment of discogenic pain, a major symptom of IVDD, by targeting the underlying inflammatory mechanisms rather than merely managing symptoms. Secondly, the modulation of the inflammasome pathway through a targeted approach, such as sLN treatment, offers a potential strategy for preserving disc integrity and function over time. This represents a significant advancement over current treatments that primarily focus on symptomatic relief. Moreover, considering the complexity of IVDD, combining sLN with other therapeutic modalities (e.g., physical therapy, NSAIDs, or newer biological agents) could be investigated to determine a possible synergistic effect that could enhance overall treatment efficacy.

Finally, this study sets the stage for further investigations into the therapeutic potential of peptides like sLN in not only IVDD but also other inflammatory and degenerative diseases [60,61]. Future research could explore the broader applicability of this approach, possibly extending it to diseases like osteoarthritis and rheumatoid arthritis where inflammasome activity is also detrimental [62,63,64,65,66]. Furthermore, our study underscores the importance of targeting specific molecular interactions within the inflammasome signalling pathways. The interaction between sLN and CD14 not only sheds light on a novel regulatory mechanism but also suggests that similar peptides or small molecules could be designed to target other components of the inflammasome pathways, providing a basis for developing new anti-inflammatory therapies. Expanding the investigation to include other models of degenerative and inflammatory diseases could uncover additional uses for sLN. Its effects on other inflammasome-related conditions, such as osteoarthritis or rheumatoid arthritis, would be valuable to explore.

This study has several limitations. Firstly, it principally used in vitro and an in vivo rabbit model to assess the effects of sLN on inflammasome activity. While these models provide valuable insights, they do not always perfectly mimic human pathophysiological conditions. Differences in disc cell behaviour and immune responses between humans and rabbits might influence the translatability of the findings. Secondly, the effective concentrations of sLN and the method of its delivery were optimized for experimental conditions. In clinical settings, achieving and maintaining effective therapeutic concentrations at the target site will be important. Thirdly, this study focused on the acute response of disc cells and macrophages to sLN treatment. The long-term effects of repetitive administration of sLN remain unknown, which is crucial for chronic conditions like IVDD. Finally, while this study identified interactions between sLN and CD14, the downstream signalling pathways and cellular effects following this interaction need further clarification to fully understand the mechanism of action. By addressing these limitations, we can better understand the potential of sLN as a novel therapeutic agent and its role in the management of IVDD and other related inflammatory conditions. These efforts will be crucial in moving from experimental insights to practical, clinically therapies.

In conclusion, our study has significantly advanced the understanding of inflammasome regulation within IVD cells, highlighting the potential of sLN peptide as a modulator of inflammatory processes associated with IVDD. We have demonstrated that sLN interacts specifically with the CD14 receptor to effectively inhibit the activation of key inflammatory markers such as NFκB, caspase-1, and IL-1β. This interaction leads to a reduction in pro-inflammatory cytokine production, thereby mitigating the inflammatory cascade that contributes to disc degeneration and associated pain. While further research is needed to translate these findings into clinical applications, our study provides a strong foundation for the development of new treatments aimed at the molecular mechanisms of disease progression in IVDD. This approach not only has the potential to improve the quality of life for patients but also offers a more sustainable and effective management strategy for chronic spinal conditions. To validate the therapeutic potential of sLN, future studies will aim to transition from preclinical models to human clinical trials. This will help ascertain the efficacy and safety of sLN in human subjects with IVDD.

## Figures and Tables

**Table 1:** Primer sequences for human genes.

Human Genes	Primer Sequence
*h-IL1β*	**F:** 5′-ACCTATCTTCTTCGACACATG-3′ **R:** 5′-ACCACTTGTTGCTCCATATCC-3′
*h-GAPDH*	**F:** 5′-TGTAAAACGACGGCCAGT-3′ **R:** 5′-CAGGAAACAGCTATGACC-3′
*h-NLRP3*	**F**: 5′-GGGTCTCCTCTCTCATCCA-3′ **R:** 5′-AGCCTCCTGAACCAGGTCTTA-3′
*h-PYCARD*	**F:** 5′-ACATCCAGCAGGCTAGAAG-3′ **R:** 5′-AAGATGCGGAAGCTCTICAGTT-3′
*h-Caspase-1*	**F:** 5′-AAAGAAAGGTCCAATAGCCAGTTT-3′ **R:** 5′-CTTCTTCTGGTCAGTGCAGAC-3′

**Table 2:** Primer sequences for murine genes.

Murine Genes	Primer Sequence
*m-IL1β*	**F**: 5′-TGGACCTTCCAGGATGAGGACA-3′ **R:** 5′-GTTCATCTCGGAGCCTGTAGTG-3′
*m-ARG1*	**F:** 5′-TGTAATGAAAGACGGCACACC-3′ **R:** 5′-TCTTCTTTGGGTATTGCTTGG-3′
*m-CD86*	**F:** 5′-ACGTATTGGAAGGAGATTACAGCT-3′ **R:** 5′-TCTGTCAGCGTTACTATCCCGC-3′
*m-CD80*	**F:** 5′-CCTCAAGTTTCCATGTCCAAGGC-3′ **R:** 5′-GAGGAGAGTTGTAACGGCAAGG-3′
*m-IL10*	**F:** 5′-CGGGAAGACAATAACTGCACCC-3′ **R:** 5′-CGGTTAGCAGTATGTTGTCCAGC-3′
*m-TNFA*	**F:** 5′-ATGAGCACAGAAAGCATGATCCG-3′ **R:** 5′-CCTTGTCCCTTGAAGAGAACCTG-3′

## Data Availability

All research was conducted in Dr. Antoniou and Dr. Mwale’s lab at the Lady Davis Institute and the data were shared through the LDI internal network.

## Supplementary Materials

The following supporting information can be downloaded at: https://www.mdpi.com/article/10.3390/biom14101312/s1, Original Western blots.
