# Supplementary material for: Short Link N Modulates Inflammasome Activity in Intervertebral Discs Through Interaction with CD14
Source: Biomolecules. 2024 Oct 16;14(10):1312. doi: 10.3390/biom14101312 (PMC11505976; doi:10.3390/biom14101312)
Supplement: Supplementary file 1 [file biomolecules-14-01312-s001.zip › biomolecules-3135000-supplementary.pdf]

Supplemental Material – Western Blots

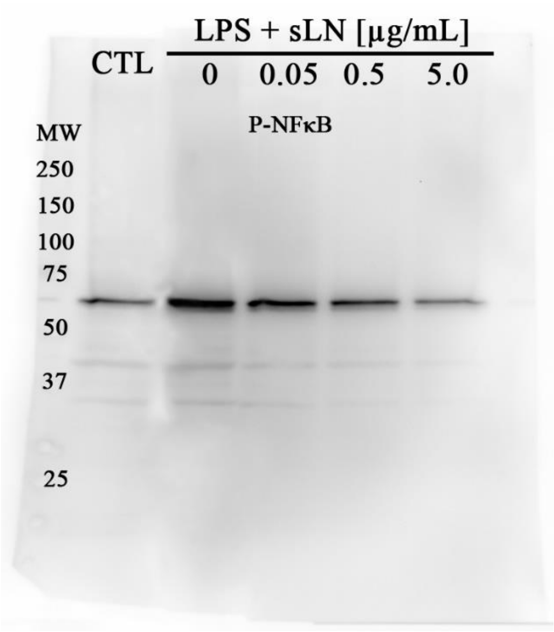

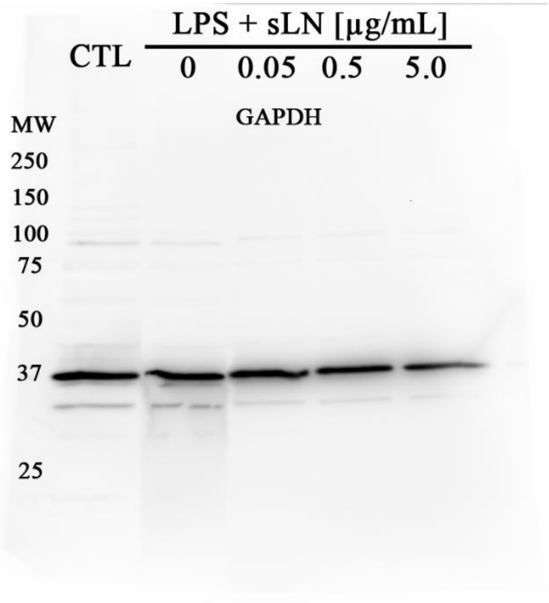

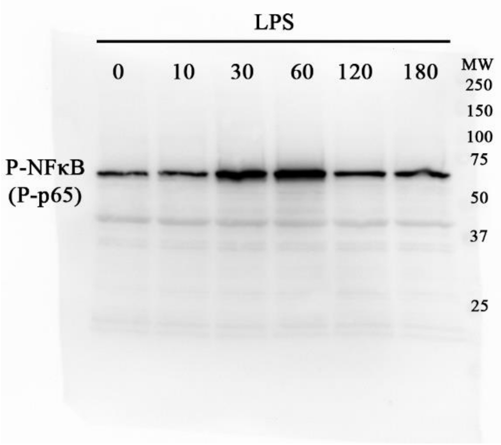

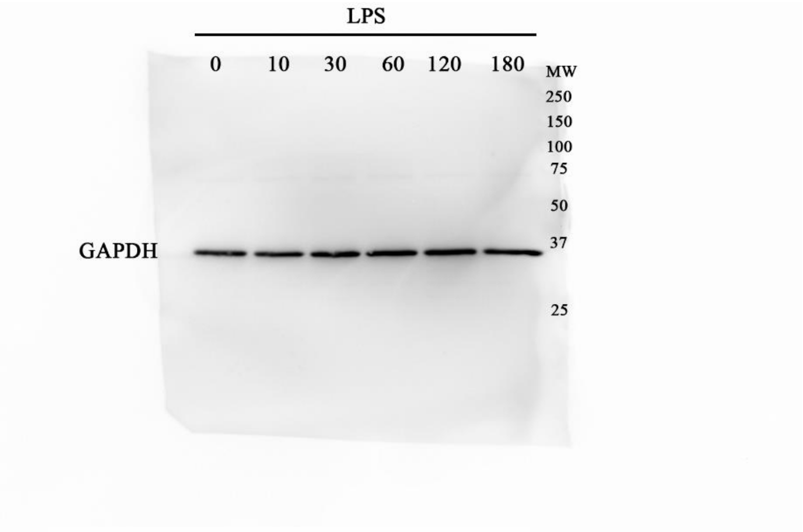

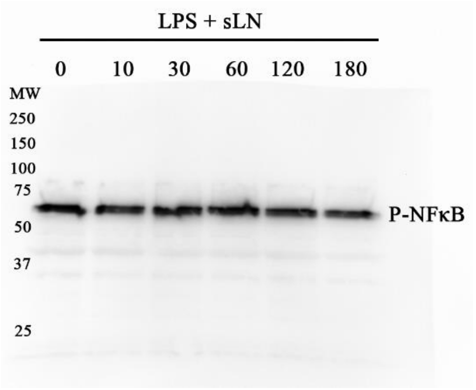

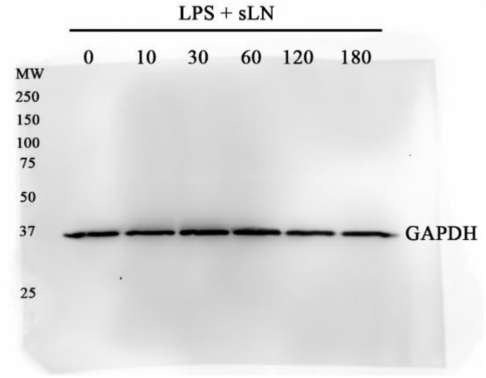

Figure 2. Evaluation of NF-κB Activation in Human Nucleus Pulposus Cells Treated with sLN and LPS. A) Human nucleus pulposus (NP) cells were treated with LPS alone or in combination with sLN with the indicated concentrations (0.05, 0.5 or 5.0 μg/mL) for 45 min. Western blotting was performed to detect activation of P-NF-κB. Blots were normalized to GAPDH for loading by densitometry and calculated as a fold increase over control.

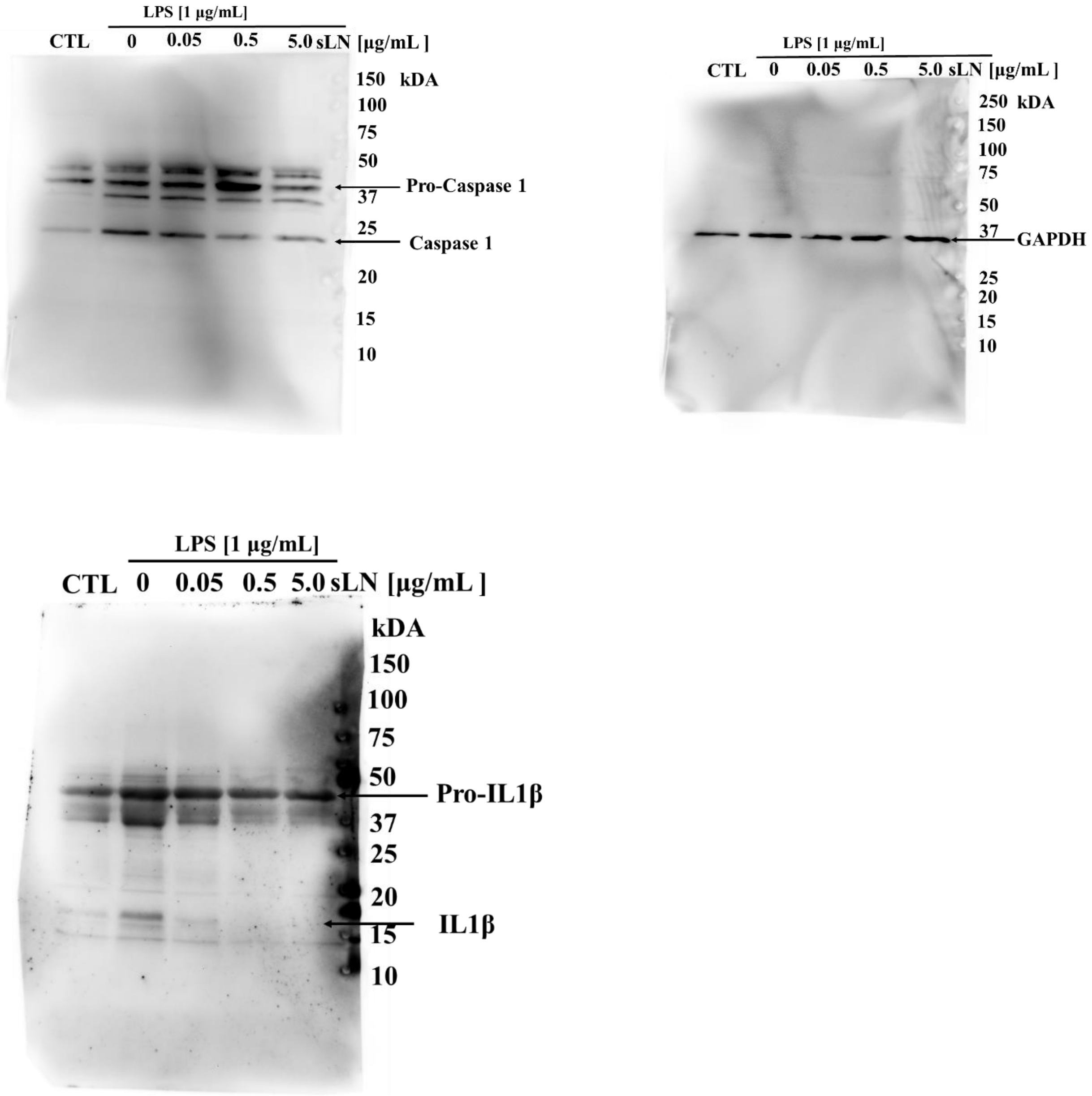

Figure 4. Assessment of Caspase-1 Activation and IL-1β Secretion in hNP Cells. HNP cells were treated with LPS alone or in combination with sLN at varying concentrations (0.05, 0.5 and 5.0 μg/mL) for 48 hours. A) Western blotting of Caspase-1 demonstrating Pro-Caspase-1, Caspase-1 and GAPDH as loading control.

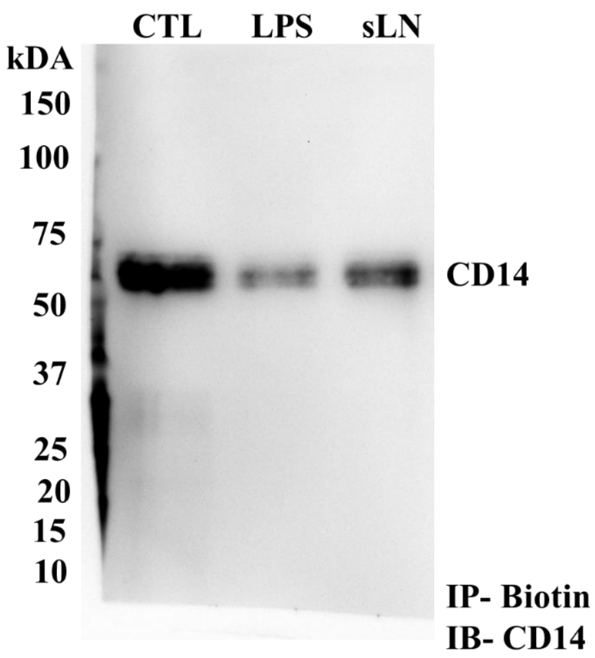

Figure 6. Interaction and Immunoprecipitation of sLN and CD14. A) Schematic on the co-immunoprecipitation of CD14 and sLN.
